# Changes in postural sway during upright stance after short-term lower limb physical inactivity: A prospective study

**DOI:** 10.1371/journal.pone.0272969

**Published:** 2022-08-24

**Authors:** Takuro Ikeda, Makoto Takano, Shinichiro Oka, Akari Suzuki, Kensuke Matsuda

**Affiliations:** 1 Faculty of Medical Sciences, Department of Physical Therapy, Fukuoka International University of Health and Welfare, Fukuoka City, Fukuoka, Japan; 2 Department of Rehabilitation, Kouichi Orthopaedic Clinic, Meguroku, Tokyo, Japan; 3 Faculty of Rehabilitation, Department of Physical Therapy, Reiwa Health Sciences University, Fukuoka City, Fukuoka, Japan; 4 Faculty of Health Sciences at Fukuoka, Department of Physical Therapy, International University of Health and Welfare, Okawa City, Fukuoka, Japan; University of Innsbruck, AUSTRIA

## Abstract

Previous studies have reported that motor behavior is affected by short-term physical inactivity using cast immobilization; however, the effects of inactivity on postural sway are not well-understood. This study aimed to investigate the effects of short-term lower limb disuse on postural sway in the upright position after cast removal. Twenty-two healthy young adults were enrolled, and each participant’s lower limb on one side was fixed with a soft bandage and medical splint made from metal and soft urethane for 10 h. Fluctuations in the center of pressure (COP) were measured before and after immobilization; the total trajectory length, mean velocity, COP root mean square (RMS) area, mean medial-lateral (M-L) COP, and mean anterior-posterior (A-P) COP were selected as evaluation parameters. Compared with the postural sway before cast application, we noted an increase and shift (from the fixed to the nonfixed side) in the postural sway after cast removal. Our results therefore suggest that short-term disuse may cause acute changes in COP movements during quiet standing. Moreover, patients may maintain their standing posture by adopting a compensatory strategy involving lateral control, similar to individuals with stroke and patients who have undergone total knee arthroplasty.

## Introduction

The human body is not completely stationary, and the bipedal upright stance is unstable owing to a small base of support and high center of mass [1]. To maintain a stable standing position and to stand with as little sway as possible, the center of pressure (COP) must be continuously corrected. Disease (i.e., stroke) and injury (i.e., fracture) often impair the stability of the standing position [2–4]. Several studies have suggested that a decline in postural control leads to balance deficits and increases the risk of falling [5–7]; therefore, for several decades, many researchers have focused on the mechanisms underlying postural instability. Understanding the factors that can impact postural control is necessary for developing more effective rehabilitation strategies.

Body joints are actively involved in maintaining posture; furthermore, for the ability to stand with as little sway as possible, multiple joints of the body must be coordinated to increase stability [8,9]. However, when part of the body is artificially rendered inactive, it may not be able to perform smooth, efficient, and precisely coordinated movements. Several studies have examined the kinematic effects on the upper limb after physical inactivity imposed by casts, splints, bandages, cotton slings, and similar devices. Moisello et al. observed changes in interjoint coordination and drifting of the movement onset point during upper limb movement [10], while Scotto et al. reported that pointing movements on a personal computer delayed movement time while maintaining accuracy [11]. Other studies have shown an increase in the total duration of reaching movements and modified anticipatory postural adjustments of the elbow and shoulder [12,13]. Interestingly, these results were obtained in healthy adults and were induced by physical inactivity of only 10–24 h.

As demonstrated in previous studies, interjoint coordination may therefore change after a short period of physical inactivity, resulting in a different strategy to achieve a stable standing posture. However, to the best of our knowledge, although some studies have examined body sway in the upright position during joint restraint, none have performed such evaluations after physical inactivity [14,15]. In clinical practice, rehabilitation for stroke in the chronic phase of recovery includes constraint-induced movement therapy (CIMT), which involves restraint of the unaffected limb and forced use of the affected limb [16]. This technique is performed for a limited amount of time during the day, and there are reports that several weeks of intervention can improve balance [17,18]. Knowledge concerning the immediate effects of physical inactivity may not only clarify the acute adverse effects of inactivity, but also contribute to our understanding of the mechanisms by which CIMT results in recovery.

This study aimed to investigate the effects of 10 h of lower limb physical inactivity on postural sway in an upright stance after cast removal (when compared with postural sway before cast application) in healthy adults, using the minimum duration of restraint mentioned in a previous study of upper extremity kinematics as a reference [10]. We hypothesized that postural sway would be greater after cast removal than before cast application.

## Methods

### Participants

The study was approved by the ethics committee of the International University of Health and Welfare (16-Ifh-041) and was performed in accordance with the Declaration of Helsinki for human participants, and there were no foreseeable risks to the participants. No personal information was collected; written informed consent was obtained; and the participants themselves provided background information.

The required total sample size was calculated using G*Power software (version G*Power 3.1.9.7) [19] with the following parameters: Wilcoxon’s signed rank test, power of 0.95, significance level of 0.05, effect size of 0.96. This resulted in an estimated minimum sample size of 17 participants. Therefore, 22 healthy young male adults (mean age ± standard deviation [SD], 21.2 ± 0.8 years; height, 170.7 ± 5.3 cm; weight, 64.0 ± 6.7 kg), all of whom were enrolled at the International University of Health and Welfare, participated in the current study. Females were excluded because postural sway is greater during ovulation, during which ankle muscle tension and stiffness are decreased, and these factors have been reported to be associated with one another [20]. None of the participants had a history of significant medical, neurological, or psychiatric diseases. Potential participants with a history of ligament injuries or lower limb fractures were excluded.

### Experimental conditions

For each participant, the lower limb on one side was fixed with a soft bandage and medical splint made from metal and soft urethane, from just above the knee to the proximal phalanx with the ankle held in a neutral position. The non-dominant leg was selected as the immobilization side in this study. The question, “Which leg do you choose to kick the ball?” was used to identify the dominant leg [21,22], enabling the selection of the symmetrical side as the immobilization leg (non-dominant leg), which performs the stabilizing role [21]. The fixed limb was the left leg in all the participants.

Participants were instructed not to move their fixed limb during the 10 h period from 09:00 to 19:00. During the 10 h immobilization, the participants followed their everyday activities while wearing the soft bandage and splint during all activities that required loading of the leg. The tip of the hallux and the other toes remained visible to control any blood circulation restriction or swelling. The participants were allowed to use their non-immobilized leg for daily living activities during the immobilization period; therefore, they were provided with crutches and trained in their use before cast application. During immobilization, the participants were regularly checked for numbness or tingling in the leg. At the end of the immobilization period, the experimenter removed the bandages and splints. The participants were subsequently instructed not to use the immobilized leg until the end of the experimental session.

### Recording and analysis

Postural sway was assessed based on fluctuations in the sway path of the COP. The COP was measured using a single force plate (Twin Gravicorder G-6100; Anima Corp., Tokyo, Japan) with a sampling frequency of 20 Hz; data were stored on a personal computer for subsequent analysis. Participants were instructed to maintain a static, upright posture on the force plate with their eyes open (EO) or closed (EC), their feet at an approximately 30° angle, barefoot, arms resting vertically at both sides of the body, and to not move or speak during the test. During EO, participants were instructed to focus on a visual target placed 2 m in front of their eyes at a visual angle of 1°. The reason for conducting the experiment with EO or EC was to demonstrate that COP sway is independent of visual information. The test started 5 s after participants stood on a single force plate to eliminate the influence of outliers. The recording time was 60 s, starting after the posture of the participants had stabilized. COP was measured before and after the 10 h immobilization period. The total trajectory length (cm), mean velocity (cm/s), COP root mean square (RMS) area (cm^2^), mean medial-lateral (M-L) COP (cm), and mean anterior-posterior (A-P) COP (cm) were selected as evaluation parameters. The total trajectory length represented the distance traveled within the two-dimensional axes in the M-L and A-P planes, observed over 60 s. The mean velocity was calculated by dividing the total distance by the trail duration trial and was the average speed at which the COP travelled. The COP RMS area was represented as a circle, with the radius calculated as the mean distance between the COP and each point of the track; the COP RMS area indicated the COP range. The mean M-L and A-P COP represented the mean of the fluctuations along the M-L and A-P axes from the theoretical center position. For the M-L axis, a negative (−) value indicated left-sided displacement (immobilization side), while a positive (+) value indicated right-sided displacement (non-immobilization side). For the A-P axis, negative (−) and positive (+) values indicated backward and forward displacements, respectively.

### Statistical analysis

Statistical analysis was performed using SPSS software (version 26.0; SPSS Inc., Chicago, IL, USA). First, a Shapiro-Wilk test was used to investigate whether the dependent variable conformed to a normal distribution, so that parametric testing could be undertaken. The results of the Shapiro-Wilk test suggested that the dependent variable was not normally distributed. Therefore, Wilcoxon signed rank tests were used to compare the results after 10 h. The alpha value was *a priori* set at p < 0.05 for all analyses. Effect sizes were examined using Cohen’s d; Cohen’s d values of 0.2, 0.5, and > 0.8 indicate small, medium, and large effects, respectively [23].

## Results

**Fig 1:**
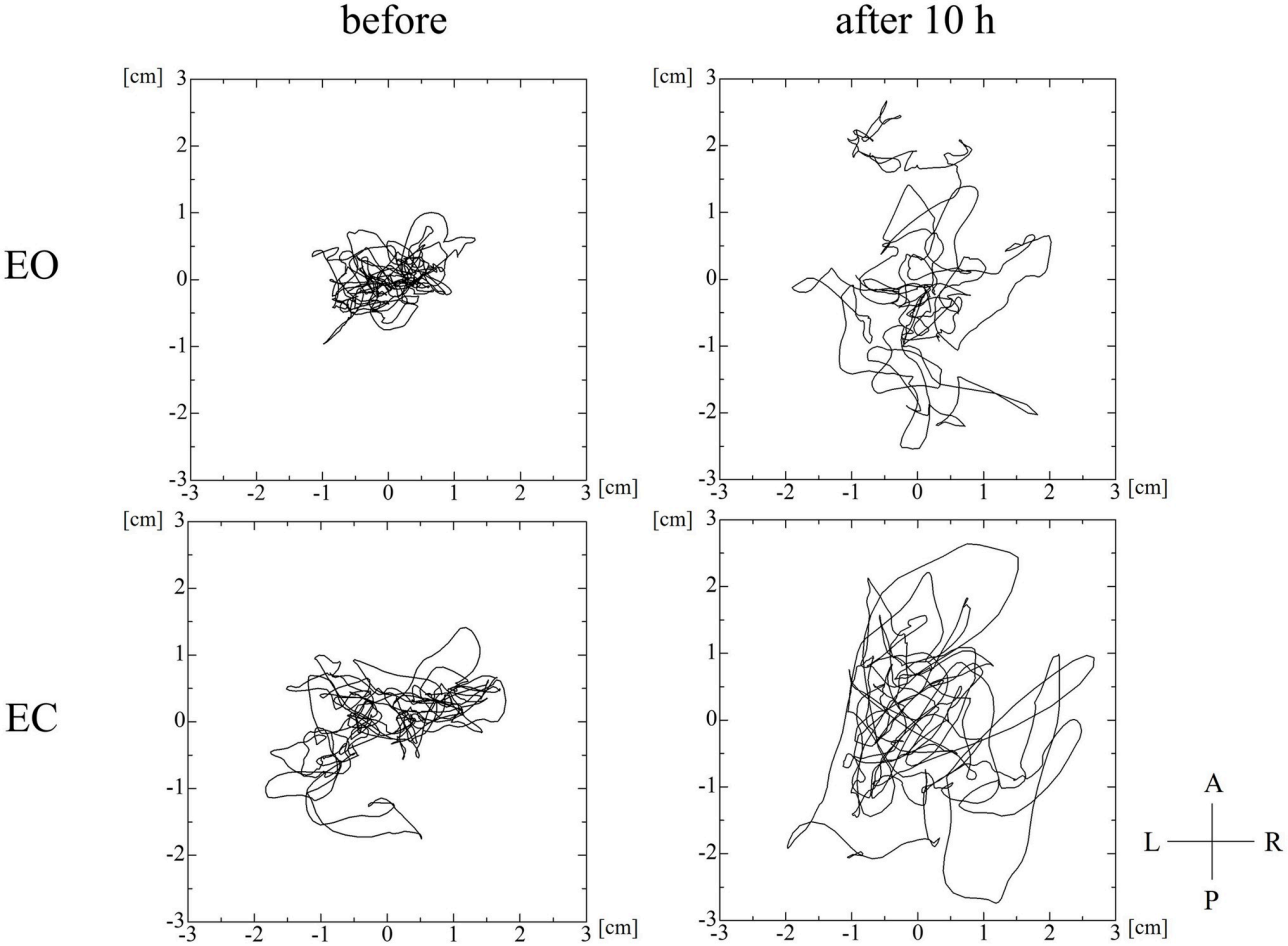
Total path followed by the center of pressure during the trials in one participant. EO: Eyes-open condition, EC: Eyes-closed condition.

There were no dropouts in this study. Fig 1 shows representative data from one participant, demonstrating the total path followed by the COP during the trials.

**Fig 2:**
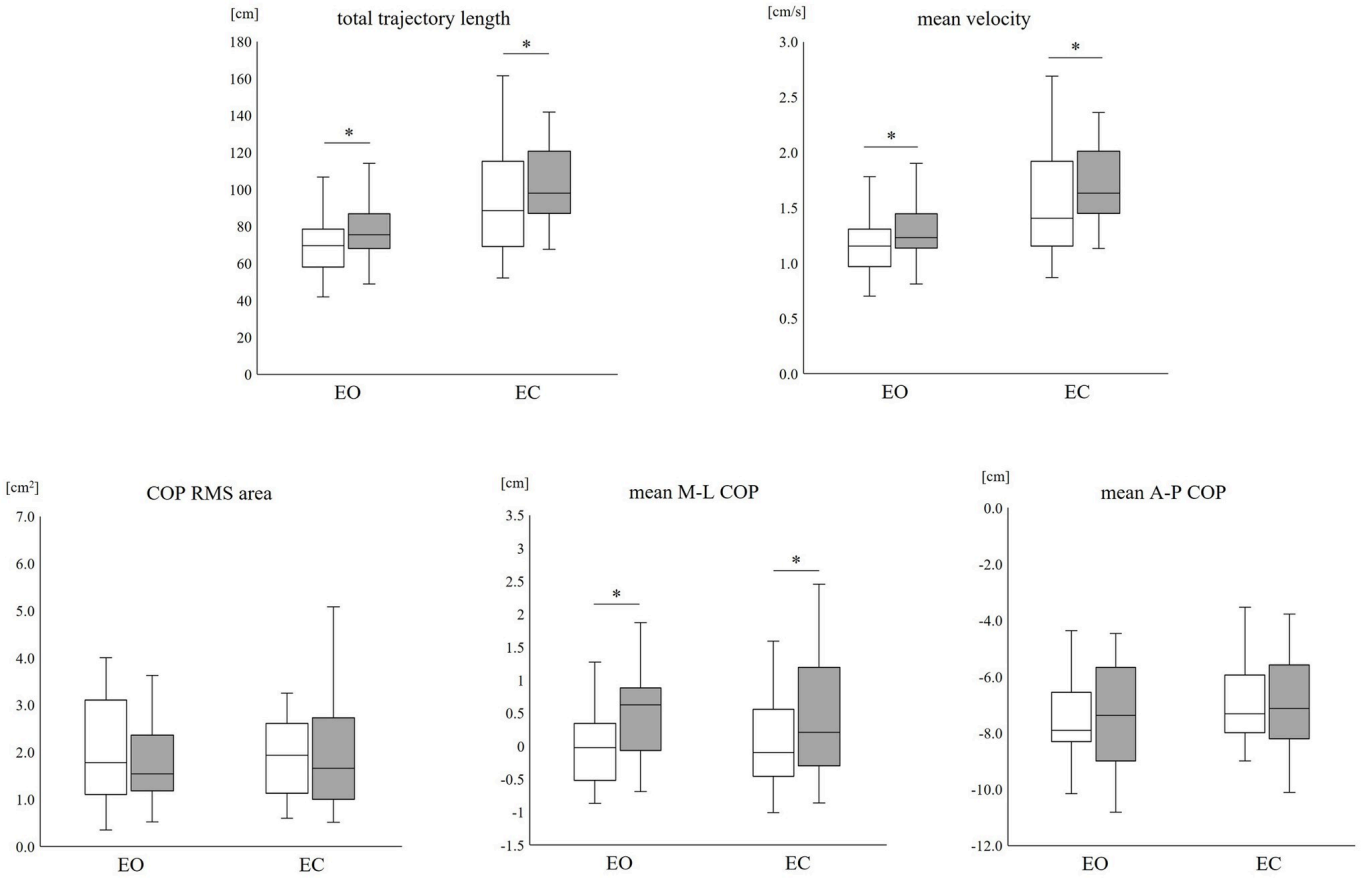
Changes in postural sway during quiet standing after 10 h lower limb immobilization. White: Before, Gray: After 10 h. *p < 0.05. A-P: Anterior-posterior, COP: Center of pressure, EO: Eyes-open condition, EC: Eyes-closed condition, M-L: Medial-lateral, RMS: Root mean square.

The changes in the COP before and after cast removal are summarized in Fig 2. There was a significant increase in the total trajectory length after cast removal when compared with that before cast application in both the EO and EC conditions (EO: p < 0.01, d = 0.53; EC: p = 0.02, d = 0.31). A similar increase in mean velocity was observed in both the EC and EO conditions (EO: p < 0.01, d = 0.09; EC: p < 0.01, d = 0.07). There was no significant increase in the COP RMS area after cast removal (EO: p = 0.68, d = 0.12; EC: p = 0.54, d = 0.15). The mean M-L COP had significantly shifted from the immobilized to the non-immobilized side after cast removal (EO: p < 0.01, d = 0.79; EC: p = 0.02, d = 0.49), while the mean A-P COP demonstrated no significant anterior or posterior displacement after cast removal (EO: p = 0.66, d = 0.11; EC: p = 0.71, d = 0.08).

In the box-and-whisker plot, the center line represents the median value, while the top and bottom boxes represent the 75th and 25th percentiles, respectively. The whiskers indicate the minimum and maximum data points in the range.

## Discussion

The present study investigated changes in postural sway during quiet standing after 10 h of lower limb physical inactivity. The COP measurement, performed while quiet standing, using a force plate provides a highly reproducibility assessment of postural balance, unaffected by circadian rhythms [24]. Therefore, it is unlikely that the postural sway is simply due to time variation. To the best of our knowledge, the current study is the first to quantify postural stability after physical inactivity. Most notably, an increase in COP movements was observed after cast removal when compared with those before cast application, and the COP had shifted from the fixed to the nonfixed side. As these significant differences were observed in healthy adults, our findings suggest that changes in COP movements during quiet standing were caused by physical inactivity rather than disease or injury.

The study that experimentally examined the effects of transient blockage of somatosensory information, the COP was altered by dermal injection of a local anesthetic into the sole of the foot [25]. The COP movements increased with increasing severity of sensory loss during standing after ischemic blockade [26]. Thus, previous studies have shown that somatosensory modalities in the lower extremities contribute to postural adjustment. In the present study, as we included healthy participants without disease, we attributed these changes to the deprivation of somatosensory and motor information caused by disuse. On the contrary, certain interpretations suggest that increased standing postural sway reflects an exploratory role by the central nervous system [27]. Thus, the increase in COP sway due to nonuse may have been an exploratory strategy of several sensory sources to keep the body in equilibrium.

In addition, several neurophysiological studies have examined the effect of limb nonuse on sensorimotor representation in healthy persons [18–32]. Facchini et al. reported a decrease in cortical excitability after 3–4 days of two-hand finger immobilization using transcranial magnetic stimulation [28]. Interestingly, several studies have demonstrated that arm immobilization for only 8–12 h induces a decrease in both somatosensory and motor evoked potentials and reduces the excitability of the somatosensory motor cortex [29–31]. These previous studies suggested that short periods of limb nonuse may trigger synaptic depression in the somatosensory motor cortex. Furthermore, another study has shown that the neural activity of peripheral nerves in the somatosensory pathway changes after 10 h of cast immobilization [32]. Therefore, increase in COP movements during quiet standing may be caused by modifications in limb representation in the nervous system due to physical inactivity. Reversible changes in the nervous system may inhibit the feedback or feed-forward processes necessary for posture maintenance [33].

The second main observation was that the postural sway shifted from the fixed side to the nonfixed side after cast removal. Researchers have observed that, among those with unilateral lower limb disorders, COP sway in the M-L axis is greater among patients with stroke and those who have undergone total knee arthroplasty [34,35]. Therefore, the results of this study suggest that after 10 h of cast immobilization, healthy participants may have maintained their standing posture by adopting a compensatory strategy consisting of lateral control, similar to that adopted in disease states. Conversely, the A-P axis was not affected, which may be why the COP RMS area did not expand.

Abdullahira et al. have argued that the effects of lower limb CIMT may be overestimated and that gait may be negatively affected by the asymmetry caused by unilateral limb restraint [36]. Our results may support this claim.

The present study had some limitations. First, the participants were allowed to use the non-immobilized leg for daily activities and were not restricted from full activity during the period of immobilization. Recent studies suggest that muscle fatigue increases postural asymmetry during quiet standing [37]. Thus, it is possible that the condition of the nonfixed limb affected COP sway. Second, we did not collect subjective stability outcomes in this study. Third, the effect sizes observed in this study were not large. Further studies are required to verify our findings while accounting for these limitations.

## Conclusions

The current findings indicated that after 10 h of lower limb physical inactivity, healthy adults exhibited an increase in COP movements and a shift of the COP from the fixed to the nonfixed side during quiet standing. These results indicate that short-term disuse may cause acute changes in COP movements during quiet standing. Future studies should investigate the fundamental mechanisms underlying static postural control after inactivity.

## Supporting information

S1 File Supporting information file. (XLSX)

## Abbreviations

A-P: anterior-posterior
COP: center of pressure
EC: eyes closed
EO: eyes open
M-L: medial-lateral
RMS: root mean square
